# Assessing the outcome of orthognathic surgery by three-dimensional soft tissue analysis

**DOI:** 10.1016/j.ijom.2018.05.024

**Published:** 2018-12

**Authors:** L. Vittert, S. Katina, A. Ayoub, B. Khambay, A.W. Bowman

**Affiliations:** 1 School of Mathematics and Statistics, The University of Glasgow, Glasgow, UK; 2 Institute of Mathematics and Statistics, Masaryk University, Brno, Czech Republic; 3 Glasgow Dental Hospital and School, The University of Glasgow, Glasgow, UK; 4 School of Dentistry, University of Birmingham, Birmingham, UK

**Keywords:** orthognathic surgery, facial shape, comparison with controls, asymmetry

## Abstract

Studies of orthognathic surgery often focus on pre-surgical versus post-surgical changes in facial shape. In contrast, this study provides an innovative comparison between post-surgical and control shape. Forty orthognathic surgery patients were included, who underwent three different types of surgical correction: Le Fort I maxillary advancement, bilateral sagittal split mandibular advancement, and bimaxillary advancement surgery. Control facial images were captured from volunteers from local communities in Glasgow, with patterns of age, sex, and ethnic background that matched those of the surgical patients. Facial models were fitted and Procrustes registration and principal components analysis used to allow quantitative analysis, including the comparison of group mean shape and mean asymmetry. The primary characteristic of the difference in shape was found to be residual mandibular prognathism in the group of female patients who underwent Le Fort I maxillary advancement. Individual cases were assessed against this type of shape difference, using a quantitative scale to aid clinical audit. Analysis of the combined surgical groups provided strong evidence that surgery reduces asymmetry in some parts of the face such as the upper lip region. No evidence was found that mean asymmetry in post-surgical patients is greater than that in controls.

One of the main objectives of orthognathic surgery is to normalize the patient’s dentofacial morphology by bringing them within the range of normal facial shape. Quantitative evaluation of the outcome of orthognathic surgery is therefore essential, with characterization of the normal facial shape a crucial component in achieving this.

Methods of assessing facial changes have predominantly been based on two-dimensional radiographs in the form of lateral cephalograms. Registration of pre- and post-surgical radiographs allows both bone and soft tissue changes to be quantified^1^. More recently this has been expanded into three dimensions with the introduction of cone beam computed tomography (CBCT), which allows imaging of both the hard and soft tissue structures^2^. However the use of CBCT is limited by both cost and radiation exposure concerns. At present in the United Kingdom, the most suitable reported outcome measure is based on the static inter-occlusal dental relationship^3^. This is reported as an improvement in peer assessment rating (PAR) score^4^, ^5^, ^6^.

Currently there are no standardized outcome measures to assess the soft tissue changes produced following orthognathic surgery. Previous studies have reported on soft tissue changes between pre-surgery and post-surgery facial shapes. Very few studies have reported on the outcome of surgery in comparison to a ‘normal’ reference group^7^. This distinction is important, as the elective surgical procedure, driven predominantly by aesthetics, should result in a ‘peer-acceptable’ facial soft tissue appearance. In one study, laser-scanned images of 80 adult subjects with no known facial disharmony were obtained, in order to produce an average three-dimensional (3D) facial template for the comparison of facial disproportion^8^. However, an average shape does not capture the variation that exists in a normal population. The primary aim of the study reported here was to compare groups of post-surgical patients with a sample of controls.

Systematic analysis requires structured information to be derived from raw facial surface images, to allow clear anatomical interpretation. Landmarks are a traditional and very useful starting point, but representations of the full facial surface provide much richer mechanisms for studying the shapes, and shape changes, of interest. There are several approaches to constructing surface representations, with a majority of these employing landmarks as a starting point. A common approach employs a surface template that is deformed, or warped, so that landmark positions on the template match exactly those on the surface of interest. This approach has been used to develop a dense point correspondence model, where standardized non-landmark locations on the image of interest are identified as the closest points to the corresponding positions on the warped template^9^. A further development of this approach was made by adding information on local surface curvature to guide deformation of the template and increase the quality of the match between the two surfaces in geometrical terms^10^. A different approach, applied to human faces, uses a Riemannian framework to produce a coordinate system that allows both deformation and comparison using a single elastic metric^11^. A Darcyan curvilinear coordinate system, based on the geodesic distance function from a fixed reference point, allows the facial surface to be represented as an indexed collection of level curves. In the study reported here, a new method is employed^12^. This uses surface curvature information to augment manually identified anatomical landmarks with anatomical curves and extend these into a full surface representation. The hierarchical nature of this process is illustrated graphically in Fig. 1.

**Fig. 1:**
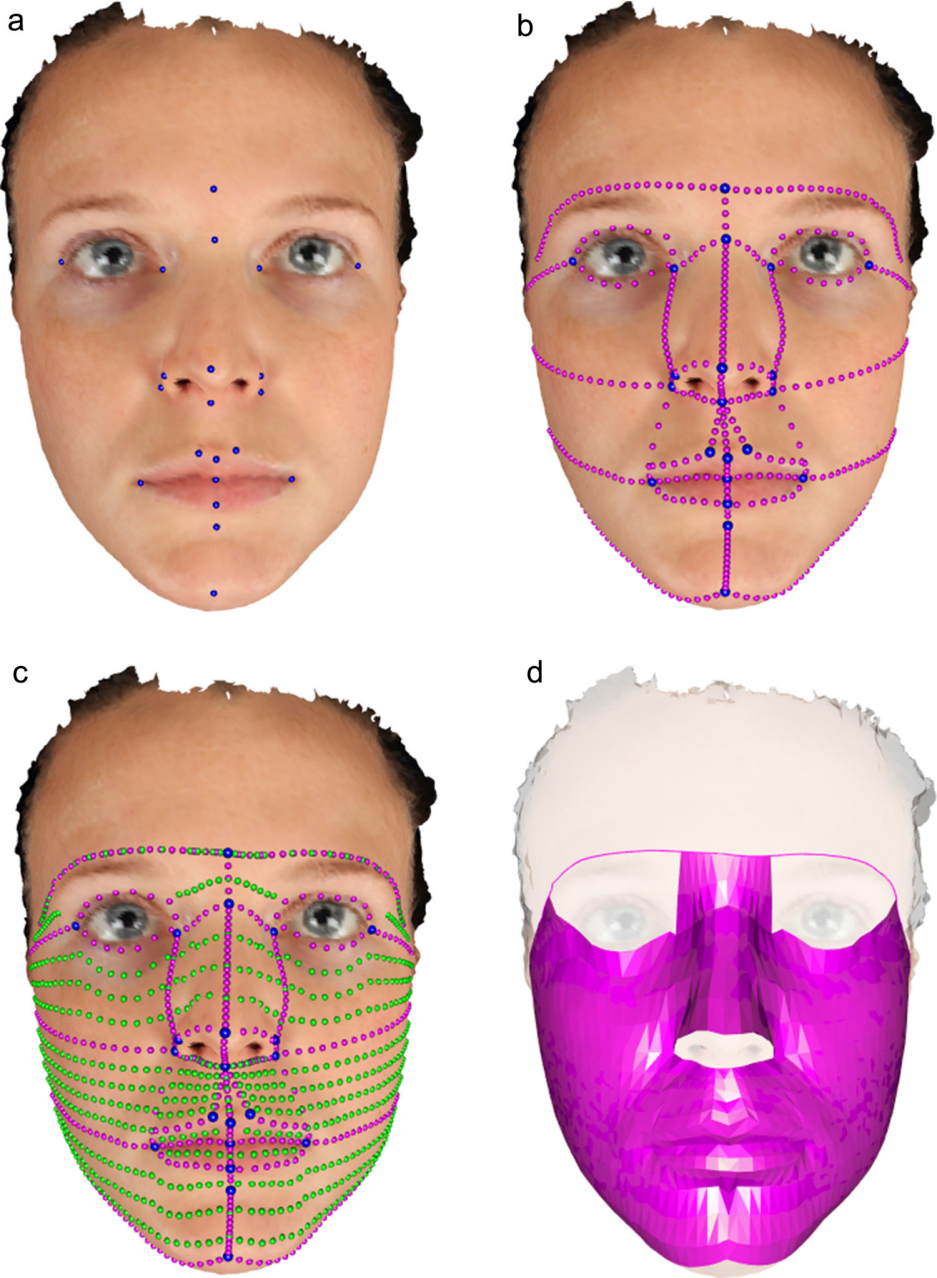
The process of construction of a facial surface model. The images from left to right show the 23 anatomical landmarks (two at the ears not visible), anatomical curves, and a full facial mesh in discretized and rendered forms.

The aim of this study was to perform a systematic analysis of images from post-surgical orthognathic patients in order to assess outcomes. The principal focus was on the comparison of postoperative facial appearance with the facial shape of controls. This was first conducted at the group level, to explore evidence of any systematic differences in outcome among patients undergoing orthognathic surgery. It was also applied at the individual level to assess the post-surgical shape of particular patients.

## Materials and methods

### Image capture

The 3D facial images of 40 patients due to undergo orthognathic surgery were captured at the University of Glasgow Dental School using a stereophotogrammetric camera system (Di3D; Dimensional Imaging Ltd, Glasgow, UK) and a capture protocol developed by the Face3D project, funded by the Wellcome Trust (see funding acknowledgements).

### Orthognathic group

All patients were diagnosed and had surgery planned using a comprehensive clinical and radiographic assessment performed by the multidisciplinary team. Patients were subdivided into three groups according to the surgical correction performed: Le Fort I maxillary advancement (*n* = 20), bilateral sagittal split mandibular advancement (*n* = 8), and bimaxillary advancement surgery (*n* = 12). The vertical maxillary position did not require correction. None of the patients exhibited facial asymmetry that warranted surgical correction. All orthognathic patients were captured shortly before surgery and at 1 year post-surgery.

### Control group

In order to obtain information on facial shape in the population at large, 169 control facial images were also captured from volunteers from local communities in Glasgow. Subjects were asked to provide basic demographic information, including age, sex, ethnic background, and any concerns or history of craniofacial anomalies, surgery, or trauma. This was used to create a control database with sufficient homogeneity to provide a focused comparison with the characteristics of the patients who had orthognathic surgery. Specifically, the following inclusion criteria were applied: (1) all parents and grandparents of White British, White Scottish, or White Irish origin (note that White Scottish is not a subset of White British); (2) no history of cleft lip/palate in the individual or a first-degree relative; (3) no history of facial accidents, facial surgery, or interest in seeking facial surgery; (4) age <53 years, to match the age range of the orthognathic patients.

In selecting control subjects, it was possible that some would exhibit facial dysmorphology that trained medical personnel might advise merits orthognathic correction. However, inclusion criteria 2 and 3 made the probability of severe clinical dysmorphology low, and the presence of a small number of cases of mild dysmorphology would not substantially affect results in a control group of this size. Retaining any such marginal cases also would have the merit of properly reflecting the facial variation present in the population at large.

**Fig. 2:**
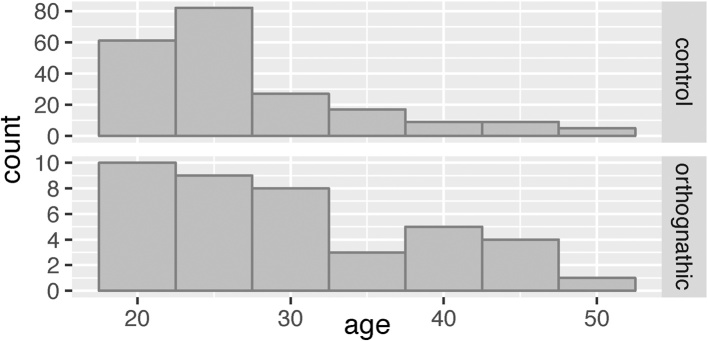
Histograms of the ages of orthognathic and control patients.

Figure 2 and Table 1 show the patterns of sex and age for the 40 orthognathic patients and 169 control subjects included in the analysis. Although the number of orthognathic cases was much smaller than the number of controls, the age distribution in the two groups was very similar.

**Table 1:** Sample sizes by sex and type of surgery, in the control and orthognathic surgery groups.

	Male	Female	Total
Orthognathic			
Maxilla, Le Fort	4	16	20
Mandible, BSSO	4	4	8
Bimaxillary, combined	5	7	12
All orthognathic	13	27	40
Controls	83	86	169

BSSO, bilateral sagittal split osteotomy.

### Landmarking

All images were manually annotated with 23 standard anatomical landmarks, as indicated in the left hand image of Fig. 1, using Landmark IDAV software^13^. Sets of anatomical curves were then produced, using the anatomical landmarks as starting points. These curves are illustrated in the second image of Fig. 1. Full surface meshes were then constructed^12^, as illustrated in the remaining images of Fig. 1.

### Statistical analysis

**Fig. 3:**
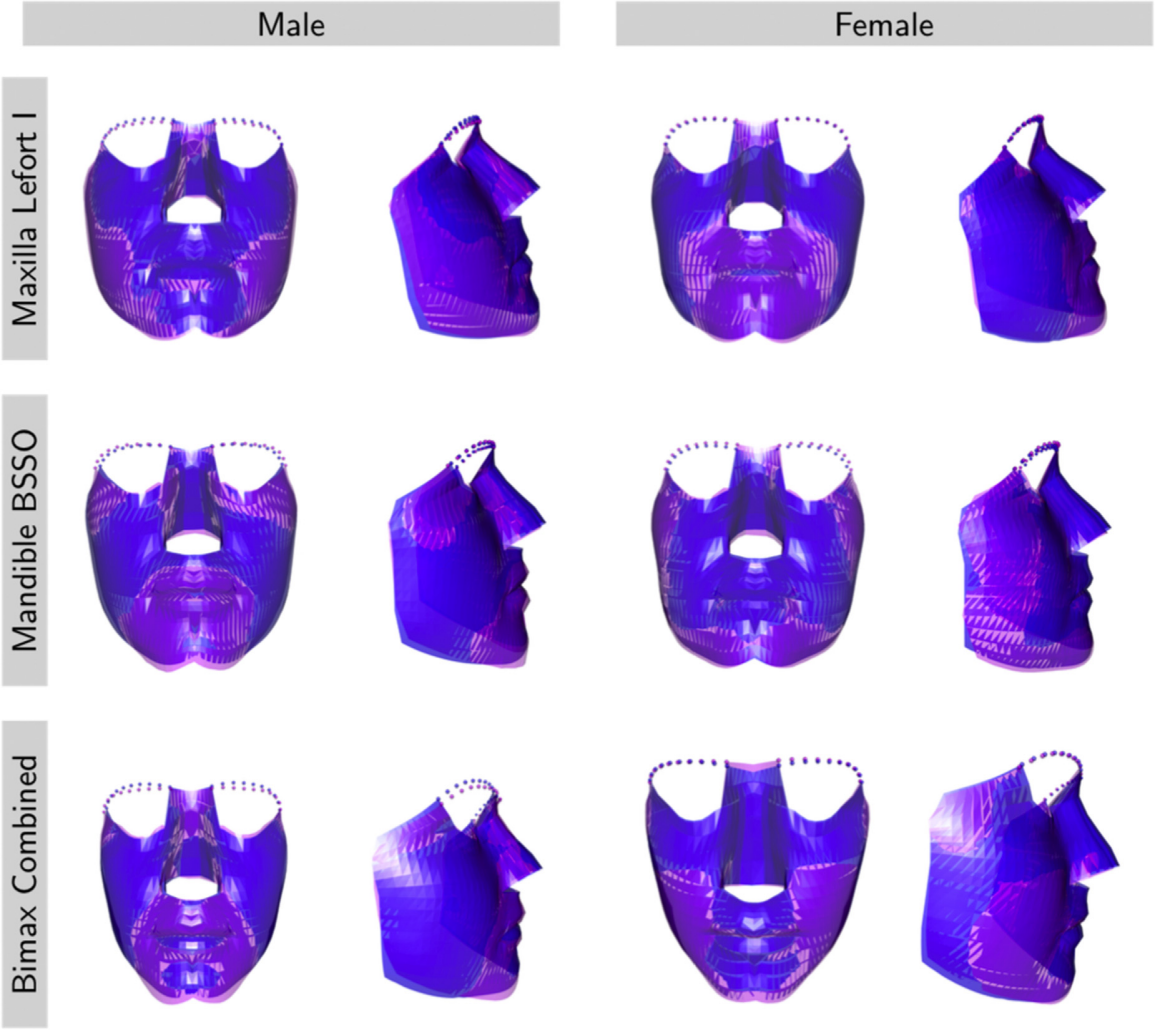
Mean shape of control subjects (blue) and post-surgical patients (magenta) for each type of surgery and each sex.

The first step in exploring differences between the post-surgical and control populations was to register the images through generalized Procrustes superimposition on the discretized mesh (shown in the third and fourth images of Fig. 1) with location, orientation, and scale effects removed. The Procrustes mean shapes could then be viewed, split by type of surgery and sex, as displayed in Fig. 3. Of course, these means cannot usefully be interpreted without considering the associated variation. Principal components analysis (PCA) is a statistical method designed for datasets where many variables are measured on each subject^14^. PCA creates a smaller number of new variables, referred to as principal components (PCs), which are able to express the bulk of the variation in the data. Each successive PC is constructed as a linear combination of the original variables, with the aim of capturing as much variation in the data as possible. The proportion of variability captured by each PC diminishes successively. In the present study, the first 10 PCs were found to capture 84% of the total variability in the data. Analysis of this small number of PCs therefore provides a very effective means of studying the patterns in the data, as an alternative to analysing the much larger number of individual variables required to define each mesh. Each PC is associated with a particular type of shape change, which can be represented and interpreted graphically.

Evidence of differences in mean shape was assessed through a two-sample Hotelling *T*^2^ test, applied to the PC scores. In order to protect against inappropriate distributional assumptions, these tests were implemented in permutation form (500 permutations). A more detailed analysis of individual PCs was also performed through *t*-tests. PCs are constructed not to identify differences between groups but the shape changes that successively capture as much variability as possible. The first few components therefore correspond to the dominant general sources of variability in facial shape, while lower components are more likely to carry the information on group differences.

With several groups to be compared to controls, a single global assessment of the evidence for change in mean shape across all groups was conducted simultaneously. However, as different surgical groups may display different departures from the shape of controls, separate analyses based on the comparison of each group against controls were applied to maximize the opportunity for these differences to be expressed.

An assessment of facial asymmetry before and after orthognathic surgery was also carried out. The resulting asymmetry scores represent the square-root of the summed squared distances between each location on the surface representation and its partner in the reflected, relabelled, and Procrustes-matched version. Several different approaches to this have been proposed^15^, ^16^, ^17^, and some allow the construction of a decomposition associated with individual features, when these are available^18^. For analysis, use of a further square-root transformation of the scores is recommended, as this transforms the scores to a scale of measurement where the variability is approximately normally distributed^18^. This additional square-root transformation was applied in the present study. Asymmetry scores were also computed for individual features, namely the lower face, the nose, and the upper and lower lips. Paired *t*-tests were used to assess the evidence for differences in pre-surgical and post-surgical mean asymmetry scores, within each surgical group.

In order to protect against the effects of multiple testing, Bonferroni adjustments (equivalent to multiplying the *P*-values by the number of tests carried out) were used when interpreting the results.

All calculations were conducted in the statistical computing environment R (R Foundation for Statistical Computing, Vienna, Austria)^19^.

## Results

### Differences in mean shape

Overall evidence for systematic differences was assessed through Hotelling *T*^2^ tests, as shown in Table 2. The evidence of differences in mean values between postsurgical and control groups was found to be unconvincing for male subjects but strong for female subjects, driven largely by the group of patients who underwent Le Fort I maxillary advancement. This was the category with the largest number of orthognathic cases (*n* = 16) and hence the strongest potential to identify differences.

**Table 2:** Differences in the means of the post-orthognathic and control populations, based on 10 components: *P*-values from the Hotelling *T*^2^ test.

	Male	Female
Maxilla, Le Fort I	0.105	0.002
Mandible, BSSO	0.066	0.058
Bimaxillary, combined	0.153	0.050
All	0.060	0.001

BSSO, bilateral sagittal split osteotomy.

**Fig. 4:**
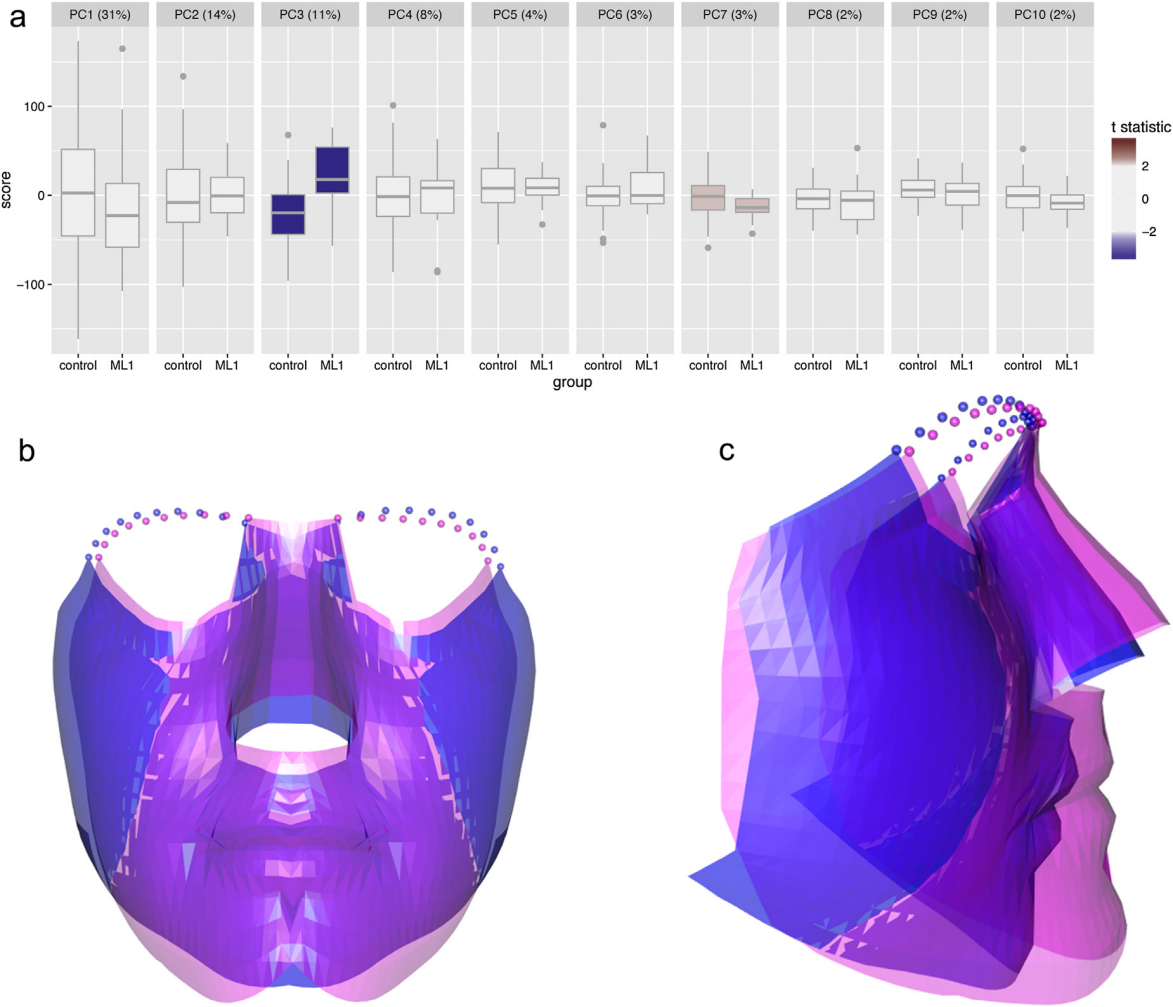
Shape change associated with the third principal component for female control subjects and maxilla Le Fort I post-surgical cases. Top: box plots of principal component scores, where the colour of the box plot indicates the size of the *t*-statistic. Bottom: suitably magnified shapes corresponding to the extremes (3 standard deviations) from a negative score (blue) to a maximal (positive) score (magenta).

The evidence is further borne out in Fig. 4, where the ranges of successive scores reflect the decreasing amounts of variability captured. The box plots for each PC are coloured to show the strength of the two-sample *t*-statistic. Strong differences between controls and post-orthognathic cases were found in the third and seventh PCs, with *P*-values of 0.001 and 0.026, respectively. Bonferroni correction for multiple testing led to a reference of 0.05/10 = 0.005, which highlights the third PC as the source of shape differences between the female patients who had undergone maxillary Le Fort I osteotomy and the control cases.

The shape change associated with the third PC is displayed in Fig. 4, using the ‘extreme’ shapes associated with 3 standard deviations from the overall mean. Positive scores in this PC correspond to protrusion of the entire midface, an effect that is mirrored by the mean shape differences in the top right-hand images of Fig. 3. This therefore suggests that a more harmonious result might be achieved for female patients undergoing Le Fort I maxillary advancement if mandibular setback surgery is performed at the same time.

### Assessment of individual patients

**Fig. 5:**
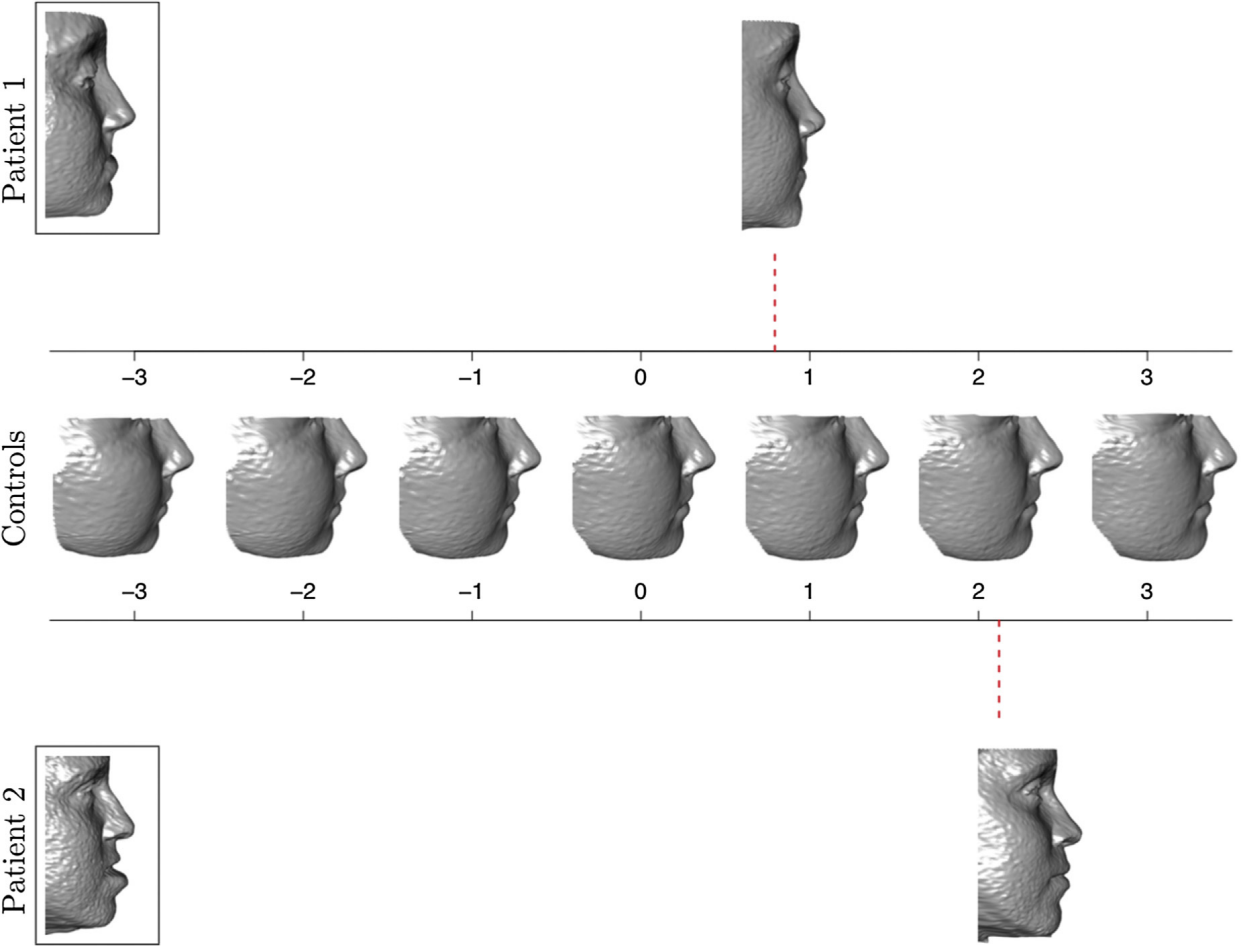
The middle row of images shows the shape change associated with the third principal component for female controls. The central image shows the control mean, while the others illustrate the shapes associated with ±1, ±2, and ±3 standard deviations of the control variation. The upper and lower rows show the pre-surgical (boxed) and post-surgical (red dashed line) shapes of two individual female patients undergoing maxilla Le Fort I surgery.

While it is of interest to identify systematic differences in shape between the post-surgical and control groups, it is also of considerable clinical interest to assess individual patients against the patterns exhibited in the control group. This is illustrated in Fig. 5. The middle row of images characterizes the variation in the control group associated with the third PC. (This is the PC associated with a systematic difference in shape between the post-surgical and control populations.) The central shape shows the control mean, while the others display the shapes associated with ±1, ±2, and ±3 standard deviations away from this mean, along the third PC. The images for ±2 standard deviations show the limits between which approximately 95% of the variation in shape lies. This can be considered to define a ‘normal range’ for control shape in this PC. The images for ±3 standard deviations indicate much more unusual control shapes.

This gives a very helpful scale against which individual post-surgical patients can be assessed. The shapes of two individual patients are shown above and below the control patterns. The two images for each patient correspond to the pre- and post-surgical shapes, and the post-surgical shape is placed on the scale at the position corresponding to the score on the third PC (now expressed in control standard deviations). For patients 1 and 2, the positions for the post-surgical shapes were 0.793 and 2.121, respectively. The pre-surgical shapes clearly reflect the inconsistency with control shape that prompted surgical intervention. For patient 1, the post-surgical shape was quite consistent with the controls, as it was found to lie within the normal range (±2 standard deviations) of the observed control variation. For patient 2, the outcome was a little unusual in the positive PC direction, with greater midface prominence than would normally be expected in controls. These two examples illustrate how surgical outcomes can helpfully be assessed in a quantitative manner.

### Asymmetry

**Fig. 6:**
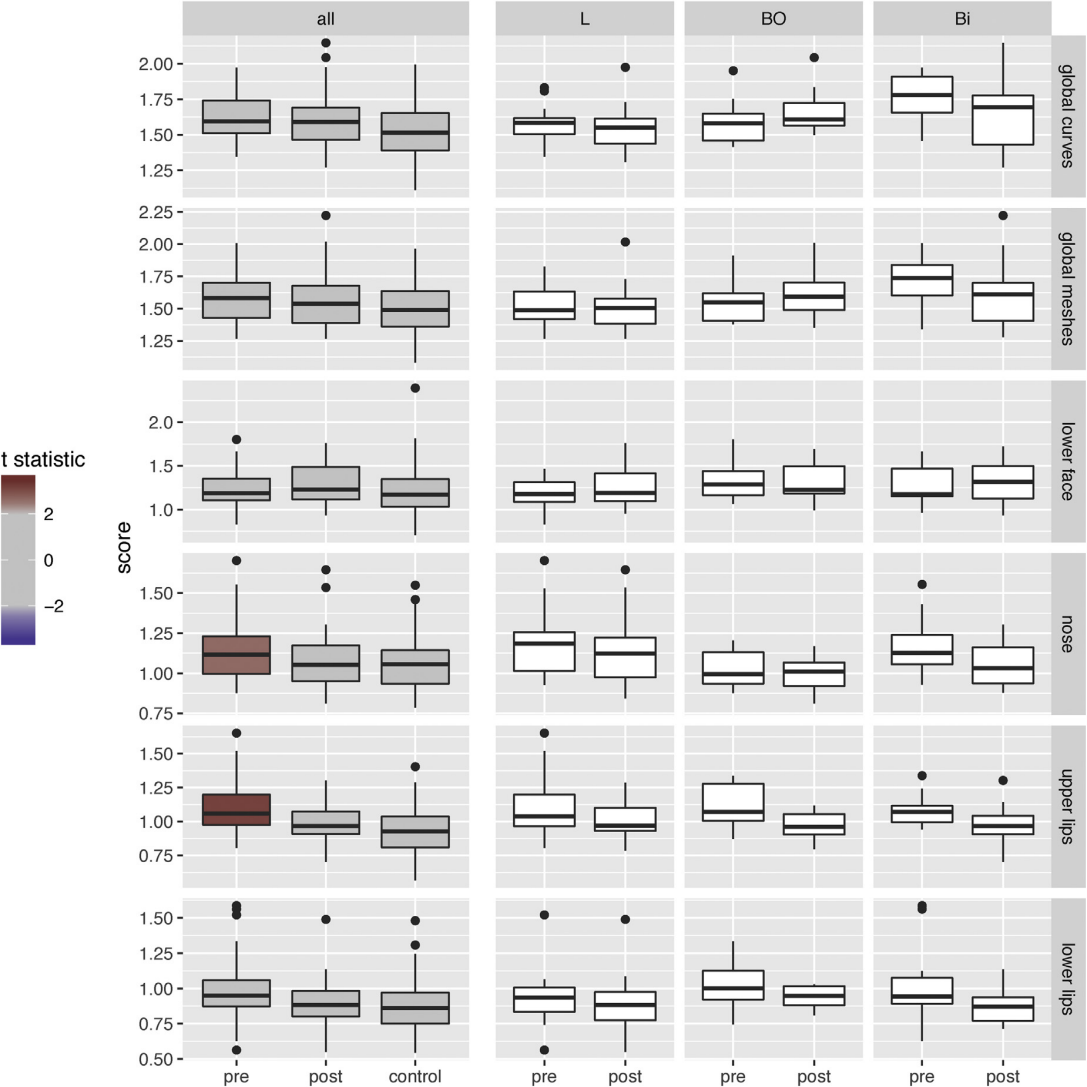
Asymmetry scores for controls together with pre-surgical and post-surgical orthognathic cases. The surgical groups are distinguished by L (Le Fort I maxillary advancement), BO (mandible bilateral sagittal split osteotomy), and Bi (bimaxillary combined surgery). In the first column of plots, the colour of the pre-surgical box plot indicates the size of the *t*-statistic for a paired comparison of means with the post-surgical group, while the colour of the post-surgical box plot indicates the size of the *t*-statistic for a two-sample comparison of means with the control group.

The asymmetry scores for controls together with pre-surgical and post-surgical orthognathic cases are illustrated in Fig. 6. The first column of plots in Fig. 6 compares pre-surgical and post-surgical asymmetry with that of controls. The remaining columns disaggregate the orthognathic cases into the different surgical groups, but the numbers involved here are too small to provide evidence of differences. The analysis is therefore concentrated on the combined surgical groups. The data are also combined across the sexes. Although male and female shape may be systematically different, there is no evidence of differential asymmetry.

The rows of Fig. 6 refer to asymmetry measurements from different parts of the face. The top two rows show scores from the whole face, constructed from anatomical curves (first row) and the full facial mesh (second row). The marked similarity here indicates that the facial curves are effective in capturing asymmetry, with little information added by the more detailed mesh. The four lower rows construct mesh asymmetry measurements from particular features: the lower face, nose, and upper and lower lips. (Note that the smaller spatial extent of these regions leads to closer Procrustes matching and hence to scores that are intrinsically slightly lower.)

In the first column of plots, which display global asymmetry, colour is used to indicate the strength of evidence for differences in means. The pre-surgical groups are coloured by the value of the *t*-statistic for a pair-wise comparison with post-surgical measurements. (Boxplots do not display the links between the pre-surgical and post-surgical pairs, but they are a useful means of displaying the overall patterns of the scores.) Strong evidence was found for a reduction in asymmetry in the upper lips (*P*-value = 0.003), but only marginal evidence for the nose (*P*-value = 0.011), with the evidence expressed in this latter value considerably diminished by the Bonferroni correction factor of a multiple of 6 for the number of tests involved. The post-surgical groups are coloured by the value of the *t*-statistic for a two-sample comparison of post-surgical cases with the controls. No convincing evidence was found here that post-surgical asymmetry was greater in comparison to controls. (The smallest *P*-value is 0.042 for the upper lips.)

## Discussion

The most significant finding of this study is the residual mandibular prognathism that was detected in the group of patients who had undergone Le Fort I osteotomy for the correction of maxillary retrognathism. These cases had the facial characteristics of a class III skeletal relationship due to the combined maxillary hypoplasia and mandibular prognathism. The decision was taken to limit the correction to Le Fort maxillary osteotomy only, in order to minimize the magnitude of surgical intervention and eliminate the potential morbidities associated with the mandibular sagittal split osteotomy, which includes permanent numbness of the lower lip secondary to damage to the inferior alveolar nerve. It was also predicted and agreed with the patients that maxillary advancement only would achieve the desired improvement in facial appearance.

The rationale of this study was to guide orthognathic surgery towards a 3D soft tissue facial shape that lies within the range of the relevant control population, with similar age, sex, and ethnic characteristics. The availability of 3D images of the soft tissue of the face provides the clinician with the opportunity to analyse, predict, and compare the outcomes in relation to a relevant control group. The results of this study suggest that there are significant differences in mean facial appearance between some post-surgical orthognathic patient groups and the corresponding control group. The results presented in this study give a clear indication of the benefits of auditing, and potentially improving, the outcome of surgery, using population-based data as an informative reference. It has been shown that although populations of white descent have strong similarities, they also display distinct morphological differences^8^. It is therefore important that morphological norms are specifically constructed for each population, in order to establish effective normative data to guide orthognathic surgical correction.

Maxillary hypoplasia is usually managed with a Le Fort I maxillary advancement, while the need for simultaneous mandibular surgery depends on the magnitude of the maxillomandibular discrepancies. In class III skeletal relationships, the bilateral sagittal split osteotomy is indicated if clinical assessment leads to a diagnosis of mandibular prognathism that requires treatment, and/or the measured discrepancy between the maxilla and the mandible is more than 10 mm. In most of these cases, mandibular setback surgery reduces the magnitude of the required Le Fort I maxillary advancement, which impacts positively on the long-term stability^20^, ^21^. In some subgroups of class III skeletal relationship, the mandible is in the correct position in relation to the nose and the upper mid third of the face. In these cases, a simultaneous advancement genioplasty is performed if mandibular setback surgery is to be carried out to deal with the marked occlusal discrepancy. This could be avoided in the pre-surgical orthodontic phase; partial dental decompensation should be considered to reduce the occlusal discrepancy and determine the magnitude of the required maxillary surgical movement. Following this protocol may eliminate the need for a simultaneous mandibular procedure.

Another important aspect that influences the decision-making process regarding the need for a simultaneous mandibular surgical procedure is the patient’s perception of their facial appearance and the desired improvements following surgery. In class III skeletal relationships mainly due to predominant maxillary deficiency, patients are focused on their paranasal hollowing, increased sclera show above the lower eyelids, the flattened upper lip, lack of upper lip fullness, and the increased nasolabial angle. Most of these perceived facial dysmorphologies will be readily addressed with a Le Fort I advancement osteotomy. The clinical features of any accompanying mandibular prognathism are generally mild and are thought to be acceptable to both the patient and the surgical team following computerized 3D prediction planning. This result is not surprising, as the aim of treatment is to restore facial harmony, which is not absolute but within a range of levels of acceptability.

When evaluating surgical outcomes, there is particular interest in asymmetry. All faces are asymmetric to some degree, but pronounced asymmetry is a feature to which the human visual system is strongly sensitive. With orthognathic patients, surgery is not conducted principally to reduce asymmetry, but these patients are more likely to exhibit asymmetry than others and so a post-surgical reduction in asymmetry is of interest. There is also the possibility that surgery may inadvertently increase asymmetry^22^. The quantification and evaluation of asymmetry is therefore valuable. It is reassuring that some evidence was found of a reduction in asymmetry between pre-surgical and post-surgical facial shape, while no evidence was found of increased post-surgical asymmetry in comparison to controls.

The results reported here are based on modest numbers of patients from a single surgical centre. The issues of sample size become more acute when the data are split by sex or by surgical group. However, the study has demonstrated the value of 3D soft tissue analysis and given a valuable insight into orthognathic surgical outcomes.

## Funding

This research was supported by a Wellcome Trust grant (WT086901MA) to the Face3D research consortium.

## Competing interests

None.

## Ethical approval

This study was approved by the West of Scotland Ethics Committee 5 (reference 10/S1001/19) for orthognathic patients and the Ethics Committee of the College of Science and Engineering, University of Glasgow, for controls.

## Patient consent

Not required.
